# Recent developments in the treatment of Parkinson's Disease

**DOI:** 10.12688/f1000research.25634.1

**Published:** 2020-07-31

**Authors:** Thomas B Stoker, Roger A Barker

**Affiliations:** 1 John van Geest Centre for Brain Repair, Department of Clinical Neurosciences, University of Cambridge, Forvie Site, Robinson Way, Cambridge, CB2 0PY, UK; 2 Department of Neurology, Norfolk and Norwich University Hospital, Norwich, UK; 3 Wellcome Trust – Medical Research Council Stem Cell Institute, University of Cambridge, Cambridge, UK

**Keywords:** α-synuclein; deep brain stimulation; drug repurposing; immunotherapies; gene therapies; neural grafting; Parkinson’s disease

## Abstract

Parkinson’s disease (PD) is a common neurodegenerative disease typified by a movement disorder consisting of bradykinesia, rest tremor, rigidity, and postural instability. Treatment options for PD are limited, with most of the current approaches based on restoration of dopaminergic tone in the striatum. However, these do not alter disease course and do not treat the non-dopamine-dependent features of PD such as freezing of gait, cognitive impairment, and other non-motor features of the disorder, which often have the greatest impact on quality of life. As understanding of PD pathogenesis grows, novel therapeutic avenues are emerging. These include treatments that aim to control the symptoms of PD without the problematic side effects seen with currently available treatments and those that are aimed towards slowing pathology, reducing neuronal loss, and attenuating disease course. In this latter regard, there has been much interest in drug repurposing (the use of established drugs for a new indication), with many drugs being reported to affect PD-relevant intracellular processes. This approach offers an expedited route to the clinic, given that pharmacokinetic and safety data are potentially already available. In terms of better symptomatic therapies that are also regenerative, gene therapies and cell-based treatments are beginning to enter clinical trials, and developments in other neurosurgical strategies such as more nuanced deep brain stimulation approaches mean that the landscape of PD treatment is likely to evolve considerably over the coming years. In this review, we provide an overview of the novel therapeutic approaches that are close to, or are already in, clinical trials.

## Introduction

Parkinson’s disease (PD) is a common neurodegenerative disease characterised by a movement disorder consisting of bradykinesia, rest tremor, and rigidity, along with postural instability, a range of other more-subtle motor features, and many non-motor features ^1^. Many of the core motor features result from the loss of a specific population of neurons: the dopaminergic neurons of the substantia nigra pars compacta, which project axons to the striatum ^2, 3^. As such, most of the current pharmacological treatment approaches for PD aim to restore dopaminergic tone in the striatum.

Whilst often effective at improving motor function, current treatments are associated with significant side effects due to delivery of dopamine to extra-striatal regions, variability in their absorption and transit across the blood–brain barrier, and the non-physiological continuous release of dopamine and its effects on the dopamine receptors within the basal ganglia ^4, 5^. Patients frequently develop cognitive problems, levodopa-induced dyskinesias, and on-off fluctuations, which we have estimated to occur in 46%, 56%, and 100% of cases, respectively, at 10 years from diagnosis based on data from our ongoing community-based incident study in PD ^6, 7^. All of these factors coupled with some of the neuropsychiatric features of PD have a significant impact on quality of life in advancing PD. Many features of PD (such as cognitive impairment and autonomic dysfunction) have a mainly non-dopaminergic basis, resulting from neurodegeneration at other sites in the central nervous system as well as the enteric and autonomic nervous systems ^3, 8^. It is often these features that have the most detrimental impact on the quality of life of patients with PD, yet treatment options remain limited for these elements of disease.

Levodopa, the precursor of dopamine, was first developed for the treatment of PD in the 1960s and continues to be the most-effective therapeutic agent for PD in 2020 ^9^. Other dopaminergic drugs have since been used, including inhibitors of dopamine metabolism as well as dopamine receptor agonists, but these are generally less well tolerated and less effective. Thus, there is an urgent need for better therapies, including disease-modifying treatments. However, the requirement for relevant pre-clinical disease models for testing such agents and the lack of robust biomarkers for diagnosing PD and the identification of prodromal disease, which would allow for treatment before significant neuronal loss had occurred, pose barriers to drug discovery.

**Figure 1.**
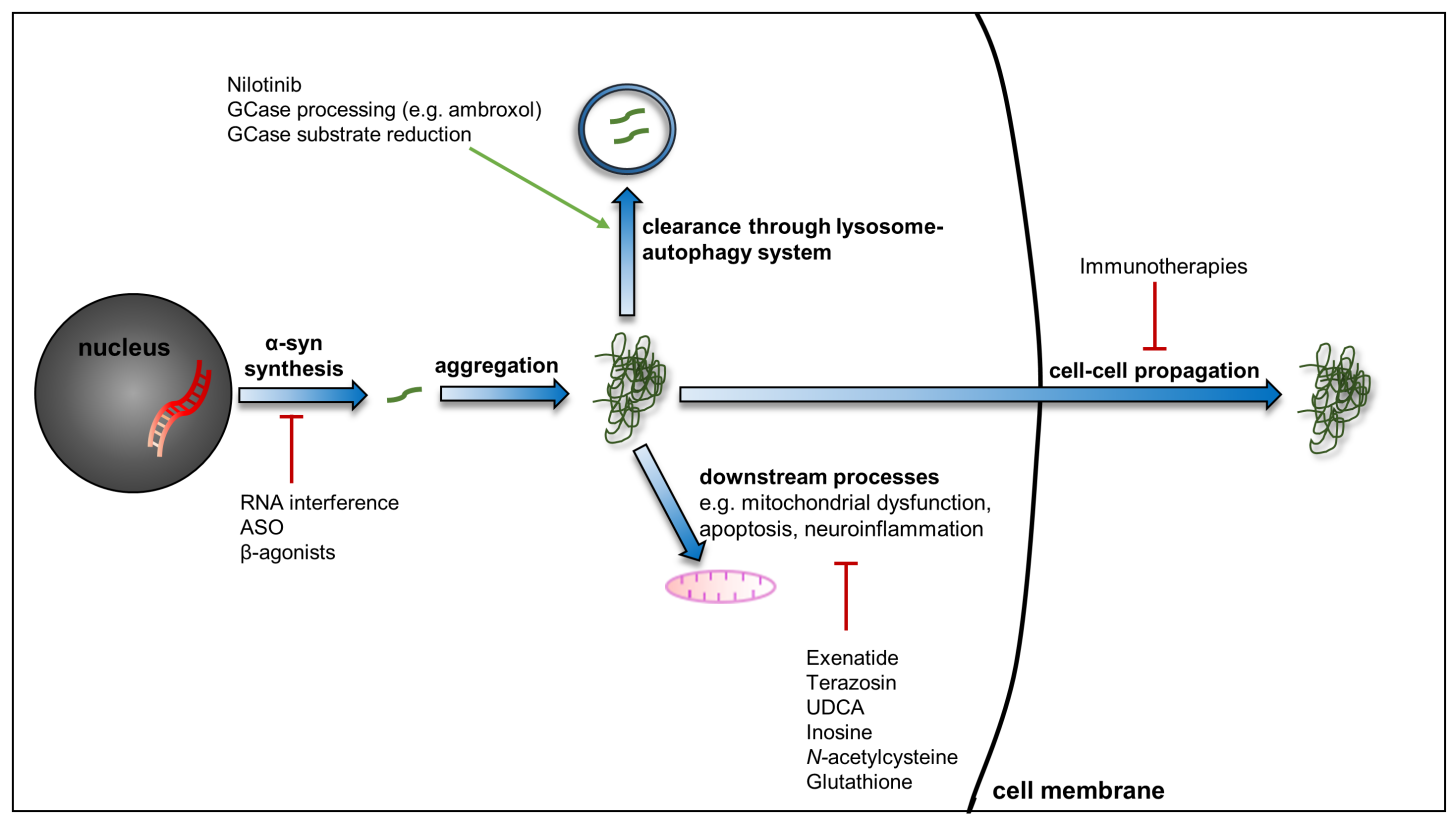
Putative disease-modifying therapies for PD. An expanding number of drugs are being considered for their ability to influence the pathogenic processes of PD. These include novel agents and technologies, such as active and passive immunisation and RNA interference techniques to limit the propagation, and synthesis, of α-synuclein. Additionally, several drugs used for other conditions are of interest for potential use in PD given their ability to influence pathways such as the lysosome–autophagy system, mitochondrial function, and neuroinflammation, for example. Abbreviations: α-syn, α-synuclein; ASO, anti-sense oligonucleotide; GCase, glucocerebrosidase; PD, Parkinson’s disease; RNA, ribonucleic acid; UDCA, ursodeoxycholic acid.

It is on this background that a number of new developments are emerging that may transform the management of PD over the coming years, and we will now discuss those that are in, or soon to be in, clinical trials ( Figure 1).

## Immunotherapies

The pathological hallmark of PD is the presence of abnormal aggregates of α-synuclein ^10^. The role of α-synuclein in PD is not clear, but it is presumed to play a central pathogenic role, as demonstrated by the fact that mutations or duplications/triplications of the gene ( *SNCA*) cause rare familial forms of PD ^11^, coupled with many independent studies showing the detrimental effects of manipulating α-synuclein in cell and animal models ^12, 13^. Potential pathogenic mechanisms of α-synuclein include dysfunction of vesicular transport, perturbations in the lysosome–autophagy system, mitochondrial dysfunction, and oxidative stress, for example ^14^. It has also been proposed that pathological forms of α-synuclein can act in a prion-like fashion, allowing pathology to spread from cell to cell, and the “strains” underlying this are now being identified ^15^. This in turn means the disease follows a pattern of pathology that results from the sequential involvement of a number of anatomical structures. All of this suggests that therapies designed to reduce levels of α-synuclein or the propagation of toxic “strains” may limit PD progression ^8^.

One experimental approach to restricting the propagation of α-synuclein is to use antibodies to target and degrade extracellular α-synuclein and thus prevent it from “infecting” neighbouring cells. Passive and active immunisation techniques against α-synuclein have been shown to convey neuroprotective effects in animal models, with the results of early clinical trials in humans starting to emerge ^14^. Other approaches to reducing α-synuclein levels include anti-sense oligonucleotide and ribonucleic acid (RNA) interference techniques to reduce its synthesis, though these remain in pre-clinical stages and are thus not discussed in detail here ^16– 18^.

A humanised monoclonal antibody targeting the C-terminus of aggregated α-synuclein (prasinezumab or PRX002, Prothena) has been shown to reduce free serum α-synuclein by approximately 97% and to be well tolerated in phase I clinical trials ^19, 20^, with a phase II trial currently underway (NCT03100149). Another antibody, BIIB054 (Biogen), targeting the N-terminal portion of α-synuclein reduces the propagation of α-synuclein pathology and improves the motor phenotype in a PD model involving injection of α-synuclein pre-formed fibrils into mice ^21^. This antibody has also been found to be well tolerated in humans ^22^ and is under investigation in a phase II clinical trial (NCT03318523).

The company AFFiRiS are approaching this problem in a different way by investigating a range of treatments consisting of α-synuclein fragments or α-synuclein-mimicking epitopes designed to induce an active immune response against α-synuclein, with phase I trials completed (NCT01568099 and NCT02267434). These products have been administered subcutaneously in early trials and seem to be well tolerated. One of these, AFFITOPE PD01A, conveyed a dose-dependent immune response to the peptide itself and to α-synuclein and is now being taken forward to phase II trials ^14^.

The use of immunotherapies to limit the propagation of PD pathology is an interesting avenue for further exploration, but important questions remain, not least the extent to which PD in the clinic is driven by protein spread. In addition, the ability of these antibodies to cross the blood–brain barrier and influence α-synuclein homeostasis in the brain is potentially an obstacle for their use in the clinic. Furthermore, neuroprotective effects of such immunotherapies appear in part to be due to intracellular effects, and their ability to enter cells may influence their efficacy. Engineered fragments (intrabodies and nanobodies) may allow for greater central nervous system penetration and entry to the cell, but these are yet to enter clinical trials ^23^. Another concern is the potential consequences of suppressing the physiological function of α-synuclein, an abundant protein whose function is incompletely understood. Suppression of α-synuclein levels in some models has been shown to be detrimental ^24– 27^, and evaluation of the long-term safety of this approach will be important. It is for this reason that some groups have sought to reduce α-synuclein through drug therapies, including the repurposing of β-agonists (see below).

## Drug repurposing

An alternative approach to limit PD pathology and disease progression is through the use of drugs that reduce α-synuclein pathology or have beneficial effects on other processes implicated in PD ( Table 1). In particular, there is a great deal of interest in drug repurposing (using established drugs for a new indication), which would potentially lead to an expedited path to the clinic, given that safety and pharmacokinetic data may already be available. Here we discuss some of the most promising agents being considered for the treatment of PD ( Figure 1).

**Table 1.** Clinical trials of putative disease-modifying treatments for Parkinson’s disease.

Drug/class	Proposed mechanism	Progress in trials
α-synuclein reduction		
β-agonists	Reduced α-synuclein transcription through acetylation of promoters and enhancers of the *SNCA* gene ^28^	Not started
Nilotinib	Inhibition of ABL tyrosine kinase activity and enhanced autophagy ^34^	Safe and tolerable but no clinical benefit in phase II trial
Terazosin	Activation of PGK1 and HSP90, increased ATP levels, and reduced α-synuclein levels ^35^	Single-centre randomised placebo-controlled trial currently enrolling patients
Mitochondrial function		
Ursodeoxycholic acid	Restoration of mitochondrial function	Randomised placebo-controlled trial currently recruiting patients
*N-*acetylcysteine	Antioxidant effect and elevation of glutathione levels ^36^	Small open-label phase II study showed no changes in indicators of oxidative damage or brain glutathione levels ^36^
Glutathione	Reduction in reactive oxygen species and free radical levels	Double-blind trial completed, with no clinical benefit demonstrated over placebo
Neuroinflammation		
Azathioprine	Modulation of peripheral immune system profile	Single-centre randomised placebo-controlled trial about to start enrolling patients
Sargramostim (G-CSF)	Induction of Treg immune responses ^37^	Phase I placebo-controlled trial completed Generally well tolerated, with reported modest improvement in UPDRS part III scores ^38^
AZD3241	Reduced oxidative stress and neuroinflammation through inhibition of myeloperoxidase	Phase 2a randomised placebo-controlled trial completed Safe and well tolerated with reduced nigrostriatal distribution of microglia ^39^
Other		
Inosine	Elevation of urate levels	Randomised placebo-controlled phase III trial halted early in 2018, with results awaited
Exenatide	GLP-1 receptor activation leading to inhibition of apoptosis, reduced microglial activation and neuroinflammation, reduced oxidative stress, and promotion of neurogenesis	Well tolerated, with improvements seen in UPDRS part III scores in randomised controlled trial ^40^ Phase III trial currently in set-up
Isradipine	Neuroprotection through blockade of L-type calcium channels in substantia nigra ^41^	Multicentre phase III trial recently completed, with no improvement in motor or quality of life outcomes
Deferiprone	Iron chelation	Phase II randomised double-blind placebo-controlled trial completed, demonstrating reduced iron content in caudate and dentate nucleus No significant clinical benefit ^42^

Abbreviations: ATP, adenosine triphosphate; G-CSF, granulocyte colony-stimulating factor; GLP-1, glucagon-like peptide-1; HSP90, heat shock protein 90; PGK1, phosphoglycerate kinase-1; Treg, regulatory T cell; UPDRS, Unified Parkinson’s Disease Rating Scale.

One class that is under consideration, but yet to enter clinical trials, is the β-adrenergic receptor agonists, given recent epidemiological and *in vitro* work demonstrating an association with reduced α-synuclein levels and risk of PD, thought to be mediated through modulation of *SNCA* transcription ^28^. Given that such agents are widely used in the treatment of reversible airway obstruction, and have been for many years, moving this to the clinic should be relatively straightforward.

Of those that have gone to clinical trials, the glucagon-like peptide-1 (GLP-1) analogue exenatide, which is used for the treatment of type two diabetes mellitus, has advanced the most. This agent has been trialled in PD patients after a similar compound (exendin-4) was found to convey neuroprotective effects in cell and animal models of nigral degeneration ^29– 31^. Several mechanisms have been proposed to mediate this effect through GLP-1 receptor activation, including inhibition of apoptosis, reduced microglial activation and neuroinflammation, reduced oxidative stress, and promotion of neurogenesis ^32^. In an initial open-label trial, exenatide was found to be safe in PD patients (though some experienced problems with weight loss), and there was an associated improvement in cognitive and motor function, which persisted after cessation of treatment ^33^. This was followed by a double-blind randomised placebo-controlled trial, which reported that once-weekly exenatide was associated with a reduction in Unified Parkinson’s Disease Rating Scale (UPDRS) motor scores in comparison to the placebo group ^40^. A multicentre phase III trial is currently in set-up, in which participants will receive weekly exenatide or placebo (NCT04232969). A pegylated form of exenatide (NLY01), which harbours enhanced pharmacokinetic properties, has also recently been taken to a phase I trial in healthy volunteers, with results awaited (NCT03672604).

Another repurposed drug that has been trialled for PD is nilotinib. This is an ABL tyrosine kinase inhibitor used in the treatment of chronic myelogenous leukaemia. ABL activity inhibits the activity of Parkin, which is important in the initiation of mitophagy, and nilotinib is proposed to enhance autophagy activity, potentially reducing the accumulation of α-synuclein aggregates ^34^. An initial phase I trial reported that the drug was well tolerated and safe, with preliminary reports of benefits on motor and cognitive function ^43^. However, there was no placebo group in this study, and some of the clinical effects observed may have been due to baseline differences between the groups and withdrawal of monoamine oxidase inhibitors in a number of subjects ^44^. Nevertheless, nilotinib has now progressed to randomised placebo-controlled trials (NCT03205488 and NCT02954978), and it appears to reduce the ratio of pathogenic oligomeric α-synuclein to total α-synuclein in the cerebrospinal fluid (CSF) ^45^. However, a recent press release for the NILO-PD trial showed that, while safe and tolerable, nilotinib did not offer any clinical benefit.

Terazosin, an α _1_-adrenergic antagonist used in benign prostatic hypertrophy, has recently emerged as a putative treatment for PD. Terazosin has been found to activate phosphoglycerate kinase-1 and the chaperone protein HSP90, which is involved in multiple intracellular stress responses ^46^. It has been shown to have neuroprotective effects in neurotoxin models of nigrostriatal degeneration in invertebrates and rodents, including after delayed administration ^35^. Additionally, terazosin reduced α-synuclein levels in transgenic mice and in neurons derived from patients with *LRRK2* mutation-associated PD ^35^. Furthermore, a retrospective epidemiological study found that people taking terazosin have a reduced relative risk of PD ^35^. These promising findings have led to terazosin rapidly progressing to a randomised placebo-controlled trial, which will involve 20 patients with Hoehn and Yahr stage 3 PD (NCT03905811). However, terazosin reduces blood pressure and can cause orthostatic hypotension, which is a problem in many patients with advancing PD and may limit its applicability in this disease.

In addition to targeting α-synuclein clearance pathways, drugs that target other intracellular pathways may be useful in PD. For example, ursodeoxycholic acid (UCDA), a drug used to treat primary biliary cirrhosis, has been found to restore mitochondrial function in cells derived from patients carrying *PARKIN* and *LRRK2* mutations as well as in invertebrate and rodent models of PD ^47– 49^. UCDA has recently progressed to a randomised placebo-controlled phase II trial, which is currently in the process of recruiting 30 patients with early PD (NCT03840005). A number of other agents are currently in, or have recently completed, clinical trials, which are summarised in Table 1.

Advances in our understanding of pathogenic subtypes of PD may allow for the targeting of specific pathogenic mechanisms in subgroups of PD patients. One such group is patients carrying *GBA1* mutations, found in approximately 5% of so-called sporadic PD patients ^50– 52^. The *GBA1* gene encodes the lysosomal enzyme glucocerebrosidase, the activity of which has been found to be reduced in PD patients without *GBA1* mutations, making it an interesting therapeutic target for a wider PD population. These mutations are associated with dysfunction of the lysosome–autophagy system, important in α-synuclein clearance ^53, 54^. Some *GBA1* mutations have been shown to lead to misfolding of glucocerebrosidase, which impairs its delivery to the lysosomal compartment, leading to perturbations in α-synuclein processing ^54^. Ambroxol, historically used as an expectorant, has recently been trialled in patients with *GBA1* mutation-associated PD, as it has been shown to facilitate the re-folding of glucocerebrosidase and increase its activity in human cells and transgenic mice with subsequent reduction in α-synuclein levels ^55, 56^. The results of the first open-label clinical trial of ambroxol in PD patients with and without *GBA1* mutations (AiM-PD) have recently been published, where the drug was found to be well tolerated over 6 months, with an associated rise in CSF glucocerebrosidase levels ^57^.

Alternatively, the glucocerebrosidase pathway may be targeted through glucosylceramide synthase inhibitors, which reduce levels of the glucocerebrosidase substrates glucosylceramide and glucosylsphingosine. Such substrate reduction therapies have been used in Gaucher disease (caused by biallelic mutations in the *GBA1* gene), but the role of these substrates in PD pathogenesis is disputed ^58^. A phase II clinical trial of a glucosylceramide synthase inhibitor (venglustat) in PD patients with *GBA1* variants is currently underway (MOVES-PD, NCT02906020).

## Targeting non-dopaminergic neurotransmitter systems

Though many of the motor features of PD are dopamine responsive, for others, such as freezing of gait and tremor, dopamine offers little benefit. It is now understood that deficiencies in other neurotransmitter systems underlie some of these features ^59^. As such, there is interest in modulating their function to treat specific dopamine-resistant aspects of PD.

One novel drug that has recently received approval for use in PD is safinamide, a drug that is proposed to have multi-modal actions. It is a potent reversible monoamine oxidase B inhibitor, conveying a benefit for the treatment of dopaminergic aspects of PD. It also modulates glutamate transmission, which may be implicated in some of the non-motor features of PD ^60, 61^. In a multicentre phase III clinical trial involving 669 patients with moderate to advanced PD, safinamide resulted in improved UPDRS motor scores, reduced off-time, and improvements in depression and communication scores ^62^. Safinamide is now becoming more widely available for clinical use, though its exact role is yet to be determined. Currently, it is most likely to be used as an adjunct to levodopa-based therapies, particularly in those who experience problematic dyskinesias and fluctuations.

Additionally, the cholinesterase inhibitors rivastigmine and donepezil have been trialled for their ability to reduce falls in PD, with promising preliminary results ^63, 64^. The noradrenaline reuptake inhibitors methylphenidate and atomoxetine are also currently being investigated for their effects on balance and gait in PD in an ongoing trial (NCT02879136). Serotoninergic neurons in the dorsal raphe nucleus have been proposed to contribute to levodopa-induced dyskinesias, and the use of serotonin agonists has been seen to reduce such dyskinesias in animal models ^65– 67^. However, their use has been accompanied by worsening of other motor features of PD in some clinical studies ^68^. However, advances in our understanding of the role of the serotoninergic system in the development of levodopa-induced dyskinesias means that there is ongoing interest in modulation of this system as a therapeutic option ^69^.

## Neurotrophic factors

Neurotrophic factors such as glial cell line-derived neurotrophic factor (GDNF) have beneficial effects on dopaminergic neurons in pre-clinical models, and there has been much interest in developing neuroprotective therapies based on these ^70, 71^.

Open-label studies of intraputaminal GDNF infusion have seen improvements in motor UPDRS scores ^72, 73^, with some evidence of restoration of the nigrostriatal pathway pathologically and on imaging ^74^. However, randomised double-blind trials have failed to recapitulate these results, including a recent study in the UK ^75, 76^. However, there has been much discussion about why these open and double-blind studies have produced such varying results, which led to a workshop in 2019 where these issues were addressed; the conclusions of which have recently been published ^77^. Whilst GDNF studies have thus far yielded mixed results, this remains an exciting experimental approach with ongoing interest. Variable results in these trials may in part be due to the involvement of patients with moderately advanced PD, inadequate follow-up times, and the large placebo effect (which is often seen in clinical trials for PD).

Neurturin, a GDNF analogue, has also been trialled in patients, with similar results to those seen with GDNF, namely promising open-label trials that have failed to translate to clinical benefit in larger trials ^78– 81^. Nevertheless, determination of the most-appropriate patients, improvement in delivery systems, and development of novel neurotrophic factor analogues mean that this approach remains an avenue of interest and is currently being explored in a new EU-funded trial looking at cerebral dopamine neurotrophic factor (CDNF, Herantis Pharma). It has recently been reported in a press release that the agent can be delivered without major side effects, although it is too early to say whether it has therapeutic benefits for patients.

## Regenerative treatments

As well as the pharmacological approaches described above, there is considerable interest in the use of cell-based and gene therapies to replace the function of the lost dopaminergic neurons. The aim of these treatments is to restore dopaminergic tone in a more targeted and physiological manner than can be achieved with current dopaminergic therapies. Several of these approaches are now entering clinical trials ^82^.

Gene therapies may be used to increase dopamine levels in the striatum through the introduction of genes that mediate dopamine synthesis. Tyrosine hydroxylase (TH) is needed for the production of the dopamine precursor levodopa, which in turn is converted to dopamine by DOPA decarboxylase, also termed aromatic L-amino acid decarboxylase (AADC). Two gene therapies involving the genes encoding these enzymes are currently undergoing clinical trials for PD.

Voyager Therapeutics have developed an adeno-associated virus (AAV) therapy containing the gene for AADC (VY-AADC). This therapy has entered a phase I clinical trial, in which 15 patients with advanced PD are receiving the treatment at three different doses. It is introduced into the putamen, with preliminary reports suggesting that the treatment is well tolerated. The effects seem encouraging, particularly given that the volume of agent delivered covers a large part of the target structure (the putamen), with corresponding increases in enzyme activity. These benefits correlated with a dose-dependent reduction in levodopa dose ^83^. A randomised sham-surgery controlled phase II trial is also ongoing (NCT03562494).

A tricistronic lentivirus vector is also currently undergoing clinical trials. This treatment consists of the genes encoding AADC, TH, and GTP cyclohydrolase 1 (which catalyses the rate-limiting step of tetrahydrobiopterin synthesis, a cofactor required for the synthesis of dopamine and serotonin). The first iteration of this treatment to enter trials, OXB-101 or ProSavin®, was assessed in an open-label phase I trial involving 15 patients with advanced PD ^84^. The treatment was well tolerated, with no serious adverse effects related to the treatment, with improvements in “off” state UPDRS scores at 12 months. However, the extent of improvement was not sufficient to make this therapy competitive. However, an improved version of this gene therapy with greater potency, OXB-102 or AXO-Lenti-PD, is currently in a two-part clinical trial in which safety will be assessed at multiple doses before progression to a randomised double-blind trial (NCT03720418).

Cell-based therapies offer another emerging approach for the targeted replacement of dopamine to treat the dopamine-dependent aspects of PD. Cell-grafting with human foetal ventral mesencephalon has been taking place since the 1980s, and whilst this has been seen to be effective in some cases with patients able to come off dopaminergic medication for sustained periods, it has become clear that logistical barriers regarding the supply of adequate tissue will prevent this from ever being a useful treatment in itself ^85– 88^. Nevertheless, a renewable source of dopaminergic cells would make cell-based therapies potentially feasible, assuming they can be shown to have sustained clinical benefits to patients.

Stem cells offer a renewable source of dopaminergic neuron progenitor cells that can be grafted into patients, and clinical trials of such products are now underway ( Table 2). Whilst controversial trials involving parthenogenetic stem cell-derived neural stem cells have been ongoing for several years ^89^, new stem cell products developed on the back of robust pre-clinical data are now progressing to trials ^82^. A clinical trial of dopaminergic progenitors derived from induced pluripotent stem cells (iPSCs) has begun (Center for iPS Cell Research and Application, Kyoto University, Japan). In this trial, seven patients will receive bilateral grafts of allogenic iPSC-derived cells. Trials involving embryonic stem cell (ESC)-derived cells are underway in China (NCT03119636) ^90^ and in set-up in the USA (NYSTEM-PD) and the UK/Sweden (STEM-PD trial). A number of other trials using ESC-derived neurons and allogenic and autologous iPSC-derived neurons are expected to commence over the next 2 to 3 years.

**Table 2.** Current and planned trials of human stem cell-derived neuronal products.

Trial	Country	Cell source	Number of patients	Status
Center for iPS Cell Research and Application	Japan	Allogenic iPSCs	7	Started
NYSTEM-PD	USA	ESCs (H9 cell line)	10	Pending decision from FDA
Chinese Academy of Sciences	China	ESCs	50	Ongoing
European STEM-PD trial	UK and Sweden	ESCs (RC17 cell line)	To be confirmed	In set-up
Fujifilm cellular dynamics international	USA	Autologous iPSCs	To be confirmed	In set-up
Allife Medical Science and Technology Co., Ltd.	China	Autologous iPS-neural stem cells	10	In set-up
Aspen Neuroscience	USA	Autologous iPSCs	To be confirmed	In development
International Stem Cell Corporation	Australia	Parthenogenetic ESC-derived neural stem cells	12	Ongoing

Abbreviations: ESC, embryonic stem cell; FDA, US Food and Drug Administration; iPSC, induced pluripotent stem cell.

## Advances in deep brain stimulation

Deep brain stimulation (DBS) is another established treatment for PD that is useful in treating dopamine-dependent motor symptoms when levodopa-induced side effects become particularly problematic. DBS involves the surgical implantation of electrodes that stimulate subcortical structures including the subthalamic nucleus and globus pallidus internus ^91– 94^. DBS offers significant improvements in motor symptoms and fluctuations in comparison to best medical therapy in some advanced PD patients, but dopamine-resistant symptoms other than tremor (e.g. gait disturbance and postural instability) respond poorly ^95^. It has also been suggested in an open-label trial that DBS is beneficial in early PD patients, with improved tremor scores and reduced development of *de novo* tremor ^96^. In addition to surgical complications, DBS strategies may cause cognitive and neuropsychiatric adverse effects as well as speech dysfunction. Novel DBS approaches, including adaptive DBS, targeting different regions, and refined intra-operative imaging techniques promise to offer improved clinical applicability and reduce the impact of adverse effects ^97^.

The pedunculopontine nucleus has recently been trialled as a new target for DBS, particularly for the gait problems seen in PD. While initial trials reported positive impacts on gait and postural instability, more rigorous subsequent trials were less promising, in part because of variability in the anatomical definition of the pedunculopontine nucleus in the human brain, suboptimal programming settings, and low patient numbers ^61, 98^. More recently, stimulation of the substantia nigra reticularis has shown promising effects on axial symptoms in preliminary studies ^99^ along with stimulation of the basal forebrain (with STN) for some of the cognitive deficits in PD ^100^. In another pilot study, thoracic spinal cord stimulation significantly reduced the frequency of freezing episodes in patients with advanced PD, with trials ongoing ^101^.

There is great interest in adaptive DBS, a system in which the stimulation delivered to the target is adjusted in response to physiological signals ^61^. This approach theoretically limits adverse effects, improves clinical response, and reduces the requirements for battery changes and the associated cost. Further work is required in identifying and validating a reliable host signal ^102^, but it is hoped that such technologies will enhance the clinical utility of DBS in the future. Non-invasive DBS techniques involving the use of external devices delivering electric fields to deep structures would circumvent the need for neurosurgery and its associated risks ^103^. One such approach that has been used more for patients with essential tremor than PD involves using magnetic resonance imaging-focussed ultrasound lesioning of discrete brain structures. Reports on the long-term efficacy of these therapies are awaited ^104^.

## Conclusion

A wide variety of experimental treatment approaches for PD have progressed towards the clinic over recent years. Many previous putative treatments have fallen by the wayside when taken to clinical trials, despite being backed up by promising pre-clinical results, emphasising the need for robust trial design. A greater understanding of the pathogenic mechanisms and anatomical basis for PD symptoms has opened up avenues for new treatment modalities, and it now seems probable that the management of PD will evolve significantly over the coming years.
